# Supplementary figures and images for: A Mutation Threshold for Cooperative Takeover
Source: Life (Basel). 2022 Feb 8;12(2):254. doi: 10.3390/life12020254 (PMC8874834; doi:10.3390/life12020254)

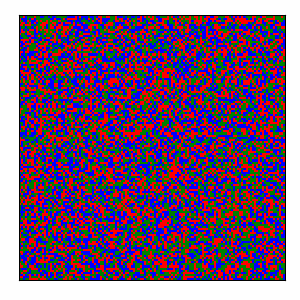

Supplement: Supplementary file 1 [file life-12-00254-s001.zip › anims/anim_000003.gif]

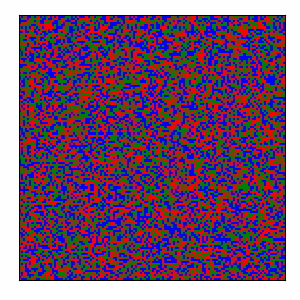

Supplement: Supplementary file 1 [file life-12-00254-s001.zip › anims/anim_000009.gif]
